# Dental Jurisprudence

**Published:** 1912-11-15

**Authors:** 


DENTAL JURISPRUDENCE. Several attempts have in the past been made to put into tangible form the law of dental relations, with more or less unsatisfactory results. The fact of the matter is that even today insufficient legal decisions of the highest courts and lack of specific legislation make it impracticable to form a satisfactory code relating to our legal relations, in the way of patient and operators rights. The newest candidate is Prof. Mikel of the University of Pennsylvania, who presents us a new work on "The laws relating to the Practice of Dentistry." Under the sponsorship of Protessor E. C. Kirk, he presents all the known law on the subject of personal rights and privileges, both statutory and judicial, as well as such logical deductions as may be fairly assumed from the general code. Professor Mikel's experience and scholarship and the evident care that he has taken in the preparation of this work, while not giving it legal authority ought to make this book of the greatest value to us in outlining at least safe principles of conduct. In so far as we have been able to examine it we have been impressed with its value and the importance of such a work for the profession. It should be in every dental library and it will undoubtedly serve as an excellent text book for college instruction. It is published in the uniform manual series of text books by Lea and Febiger, Philadelphia.

